# Arsenic Trioxide in Synergy with Vitamin D Rescues the Defective VDR-PPAR-*γ* Functional Module of Autophagy in Rheumatoid Arthritis

**DOI:** 10.1155/2019/6403504

**Published:** 2019-05-07

**Authors:** Weiyan Wang, Chunling Li, Zhiyi Zhang, Yue Zhang

**Affiliations:** ^1^Department of Rheumatology, The First Affiliated Hospital of Harbin Medical University, 23 Youzheng St., Nangang District, Harbin, China; ^2^Shenzhen Futian Hospital for Rheumatic Diseases, 22 Nonglin Road, Shenzhen, China

## Abstract

Dysregulated autophagy leads to autoimmune diseases including rheumatoid arthritis (RA). Arsenic trioxide (ATO) is a single agent used for the treatment of acute promyelocytic leukemia and is highly promising for other malignancies but is also attractive for RA, although its relationship with autophagy remains to be further clarified and its application optimized. For the first time, we report a defective functional module of autophagy comprising the Vitamin D receptor (VDR), PPAR-*γ*, microtubule-associated protein 1 light-chain 3 (LC3), and p62 which appears in RA synovial fibroblasts. ATO alleviated RA symptoms by boosting effective autophagic flux through significantly downregulating p62, the inflammation and catabolism protein. Importantly, low-dose ATO synergizes with Vitamin D in RA treatment.

## 1. Introduction

Rheumatoid arthritis (RA) is a chronic inflammatory joint disease that leads to cartilage and bone damage as well as disability [1].

Autophagy is defined as a degradation mechanism by which cells recycle cytoplasmic components. Several autophagy-related genes are involved including* Beclin1*,* microtubule-associated protein 1 light-chain 3* (*LC-3*),* p62*, and* mammalian target of rapamycin* (*mTOR*) [2–4]. The assessment of autophagic activity can be achieved by detection of the LC-3 protein. Autophagic flux is a complex process that involves transporting, binding, degrading, and recycling the cytoplasmic components. Nevertheless, increases in the levels of LC-3 can be caused by either the induction of autophagy or inhibited fusion of the autophagosomes with lysosomes; it cannot be used to monitor autophagic flux* per se*. The p62 protein serves as a link between LC-3 and ubiquitinated substrates and is degraded in autolysosomes. Thus, inhibition of the final step of autophagy correlates with increased levels of p62 and indicates impaired flux [5]. Dysregulated autophagic flux is involved in the pathogenesis of many autoimmune diseases, including RA, by regulating the organism's lifespan and cartilage homeostasis [6–9]. In addition, the activation of autophagy in immune cells is significantly associated with inflammatory parameters such as interleukin-6 (IL-6) and tumor necrosis factor-*α* (TNF-*α*), which are proinflammatory cytokines involved in the pathogenesis and progression of RA [10].

The Vitamin D receptor (VDR) is expressed in multiple cells and numerous RA and autophagy-related genes, including mTOR, are potential candidate targets of Vitamin D (Vit D) and VDR [11, 12]. It maintains “robustness” in unfavorable conditions and serves a “capacitor” in homeostasis in RA biology [13, 14]. Peroxisome proliferator-activated receptor-*γ* (PPAR-*γ*) is a primary target of Vit D [15]. In addition, both VDR and PPAR-*γ* are key autophagy regulators [16, 17] and play central roles in the development and progression of RA, supporting the possible involvement of autophagy in RA.* In vivo* and* in vitro* experiments confirmed that the PPAR-*γ* agonist could downregulate inflammatory cytokines [18, 19]. Bone erosion can be alleviated in RA patients by reducing catabolism through PPAR-*γ* pathway activation [20]. The regulatory effects of VDR and PPAR-*γ* through autophagy have been proven in tumors and other diseases [21–23]. Nevertheless, the exact role and involvement of VDR and PPAR-*γ* in RA-related autophagy remain to be defined.

The search for novel drugs that can manage more than one single age-related disease is encouraged [24]. Among these drugs, arsenic trioxide (As_2_O_3_, ATO) is recognized for treating tumors [25, 26] and autoimmune rheumatic diseases by enhancing apoptosis and inhibiting angiogenesis [27–29]. ATO has been shown to induce antitumor effects through autophagy [30]. However, the effect of ATO on autophagy in RA is unknown.

In the present study, we demonstrated for the first time that VDR, PPAR-*γ*, and LC-3 participate in a functional module of autophagy (a functional module is a group of genes that are tightly associated through multiple feedback loops) and are significantly upregulated in RA synovial tissues, although autophagic flux was unexpectedly severely impaired. Surprisingly, ATO rescued this defective functional module of autophagy in RA fibroblast-like synoviocytes (FLS) and mice with RA, and the effect was even better when ATO was used with Vit D. Hence, ATO combined with Vit D could be a potential therapeutic strategy against RA.

## 2. Materials and Methods

### 2.1. Synovial Fibroblast Culture and TNF-*α* Stimulation

RA FLS and normal human (NH) FLS were purchased from Cell Applications (San Diego, CA, USA) and maintained in a synoviocyte growth medium (Cell Applications). Cells were used for the experiments in stages 4–6. RA FLS were pretreated with 50 ng/mL of TNF-*α* for 4 h before the application of ATO and/or Vit D. Cell culture supernatants were used for enzyme-linked immunosorbent assays (ELISAs) 48 h after the addition of the treatments.

### 2.2. *In Vitro* Proliferation Assay

Cell proliferation was evaluated with a Cell Counting Kit-8 (Sigma, St Louis, MO, USA) following procedures described earlier with minor modifications [31]. Briefly, 10^4^ cells were seeded in a 96-well plate. After 24 h, different concentrations of drugs or vehicles were added with fresh medium. Cells were incubated at 37°C for 48 h. The plates were read at 450 nm. The experiments were repeated three times.

### 2.3. RNA Preparation and Real-Time Quantitative Polymerase Chain Reaction Analysis

Total RNA was extracted from FLS with TRIzol reagent (Invitrogen, Carlsbad, CA, USA) and converted to cDNA. Real-time quantitative polymerase chain reaction (PCR) amplification was performed as described previously [28]. The sequences of the primers are shown in Table 1. GAPDH was used as an internal control. All samples were measured in triplicate and the results were evaluated via the 2^−ΔΔ ct^ method [9].

### 2.4. Western Blotting

Equal amounts of proteins were obtained and separated by SDS-PAGE and transferred to a polyvinylidene fluoride membrane as described previously [28]. Specific primary antibodies were added, including anti-PPAR-*γ* (catalog no. ab41928, Abcam, Cambridge, MA, USA), anti-VDR (catalog no. 12550, Cell Signaling, Cambridge, MA, USA), anti-LC-3-I/II (catalog no. 12741, Cell Signaling), anti-p62 (catalog no. ab56416, Abcam), anti-mTOR (catalog no. ab32028, Abcam), and anti-p-mTOR (catalog no. ab109268, Abcam). The reactive bands were visualized with a chemiluminescence detection system and analyzed with ImageJ software (National Institutes of Health, USA).

### 2.5. RNA Interference

VDR knockdown was achieved by transfecting specific VDR small interfering RNA (siRNA) into RA FLS. The siRNA sequence was as follows: forward, 5′-GCUGAAGUCAAGUGCCAUUTT-3′; reverse, 5′-AAUGGCACUUGACUUCAGCTT-3′. RA FLS were plated in 12-well plates at 10^5^ cells per well with serum-free DMEM and transfected with siRNA via Lipofectamine 2000 (Invitrogen) as previously described [28]. Six hours after transfection, the culture medium was replaced by DMEM with 10% FBS. Knockdown efficiency was determined by real-time PCR and western blot analysis. The experiments were repeated at least three times.

### 2.6. Establishment of Collagen-Induced Arthritis

Specific pathogen-free 6-week-old male DBA/1J mice weighing 18±2 g were purchased from SLAC (Shanghai, China). Animal welfare and experimental procedures were carried out in accordance with the Guide for the Care and Use of Laboratory Animals and were approved by the Institutional Animal Care and Use Committee of Harbin Medical University. The collagen-induced arthritis (CIA) mouse model was established as described previously [28]. Mice were randomly assigned to different groups (*n*=6 per group): normal control group (mice without immunization and injected with saline); the CIA control group (CIA mice treated with saline, CIA-saline); the ATO-treated group (CIA mice treated with ATO at a dose of 1.0 or 2.0 mg/kg/day), Vit D (400 ng/kg/d) or Vit D (400 ng/kg/d) + ATO (2.0 mg/kg/day); and the methotrexate (MTX) group (CIA mice treated with MTX at 2 mg/kg/week as a positive control). Mice were given intraperitoneal injections from day 28 to day 41. The body weight of each mouse was recorded every other day from day 21.

### 2.7. Assessment of Arthritis Severity

To evaluate the severity of the arthritis quantitatively, the arthritis score was assessed every 2 days starting from day 21, according to a scoring system used previously [32]. In addition, body weight and the thickness of the two hind paws were measured every other day.

### 2.8. Microcomputed Tomography Imaging

To investigate the effects of ATO on the three-dimensional (3D) bone structure, we conducted an assay using microcomputed tomography (micro-CT) imaging (Quantum GX, Perkin Elmer, Waltham, USA). Mice were scanned and reconstructed into a 3D structure via micro-CT imaging 39 days after the initial collagen injection. The voxel size was 72 *μ*m, the X-ray tube voltage was 90 KV, the current was 88 *μ*A, and the exposure time was 4 min. Mean CT values of the hind paws were calculated with Caliper Analyze software (Analyze Direct, Kansas, USA) to assess bone loss.

### 2.9. Histological Analysis

Whole knee joints of the mice were collected and fixed in 10% buffered neutral formalin and decalcified in 10% EDTA for 4 weeks. The paraffin-embedded specimens were stained with hematoxylin and eosin (H&E). Histological changes were scored in a blinded manner by two independent observers based on synovial hyperplasia, joint inflammation, and bone erosion following a scoring system described previously [33, 34].

### 2.10. Immunohistochemistry Analysis

Immunohistochemistry was performed with specific antibodies for target proteins following a protocol described previously with some modifications [6]. Knee joint sections on slides were incubated with anti-VDR, anti-PPAR-*γ*, anti-LC-3, or anti-P62 (Boster, Wuhan, China) antibodies. Subsequently, the sections were stained with a polymer horseradish peroxidase (HRP) detection system (PV9001, ZSGB-BIO, Beijing, China) and visualized with a diaminobenzidine (DAB) peroxidase substrate kit (ZLI-9017, ZSGB-BIO, Beijing, China). Each section was evaluated under a microscope (DMi8, LEICA, Wetzlar, Germany) in three randomly selected areas at a magnification of 20×. Image-Pro Plus 6 (Media Cybernetics, Inc.) was used to analyze the average integrated optical density (IOD) according to a previously described protocol [35].

### 2.11. Enzyme-Linked Immunosorbent Assay

Serum samples from the mice and the cell culture supernatant were collected and stored at −80°C. The concentrations of IL-6, IL-1*β*, matrix metalloproteinase-3 (MMP-3) and MMP-13 were detected with commercial kits (Elabscience Biotechnology Co., Ltd., Wuhan, China), according to the manufacturer's protocols. All assays were conducted in triplicate.

### 2.12. Statistical Analysis

Data are represented as means ± standard error of the mean and were analyzed via Student's* t*-test or analysis of variance (ANOVA), as appropriate. All analyses were carried out in SPSS 17.0 software. Values of* p* < 0.05 were considered statistically significant.

## 3. Results

### 3.1. ATO Rescues the Defective VDR-PPAR-*γ* Functional Module of Autophagy Both* In Vivo* and* In Vitro*

**Figure 1:**
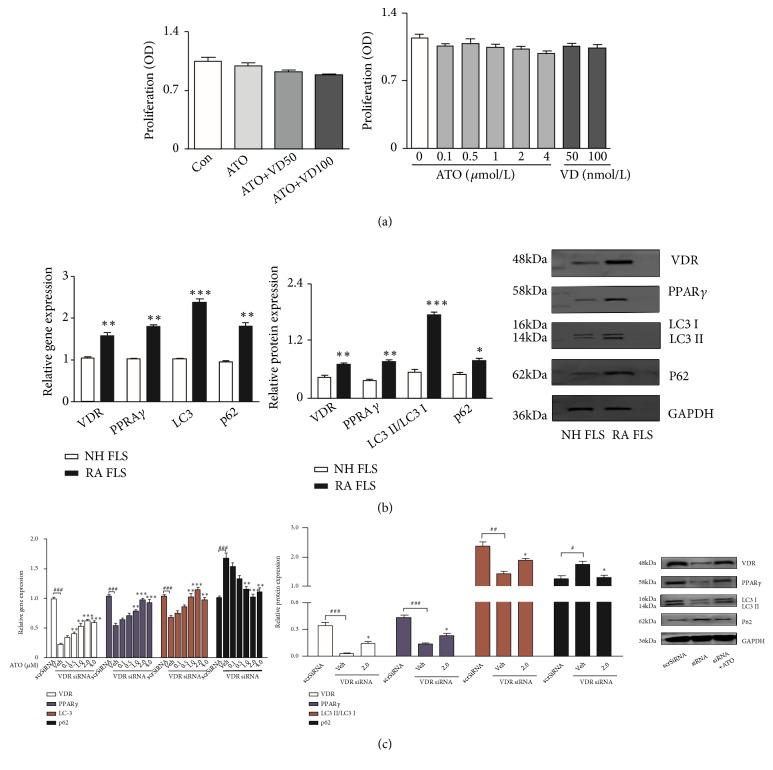
(a) After treatment with different concentrations of arsenic trioxide (ATO) (0.1, 0.5, 1.0, 2.0, or 4.0 *μ*M) or Vitamin D (Vit D) (50 or 100 nM) for 48 h (*n*=3), CCK-8 was added to rheumatoid arthritis (RA) fibroblast-like synoviocytes (FLS) and incubated for 1 h. Absorbance (optical density value) at 450 nm was detected by a microplate reader. The results showed no significant influence on cell proliferation after the administration of different concentrations of ATO and Vit D. The results are presented as the mean ± SEM. (b) Real-time PCR and western blot analysis showed significantly increased expression of the Vitamin D receptor (VDR), PPAR-*γ*, LC-3, and p62 in RA FLS (*n*=3) compared with normal human (NH) FLS (*n*=3; *∗ p*<0.05, *∗∗ p*<0.01, *∗∗∗ p*<0.001). The results are presented as means ± SEM. (c) Silencing of VDR in RA FLS significantly reduced the expression of VDR, PPAR-*γ*, and LC-3 but induced the expression of p62. Addition of ATO could reverse the effect of small interfering RNA (siRNA) (*n*=3, #*p*<0.05 ##*p*<0.01 ###*p*<0.001 versus scrambled siRNA-treated cells (SCR-siRNA); *∗p*<0.05; *∗∗p*<0.01 *∗∗∗p*<0.001 versus vehicle). Vehicle = RA FLS treated with the medium alone after silencing by VDR siRNA.

**Figure 2:**
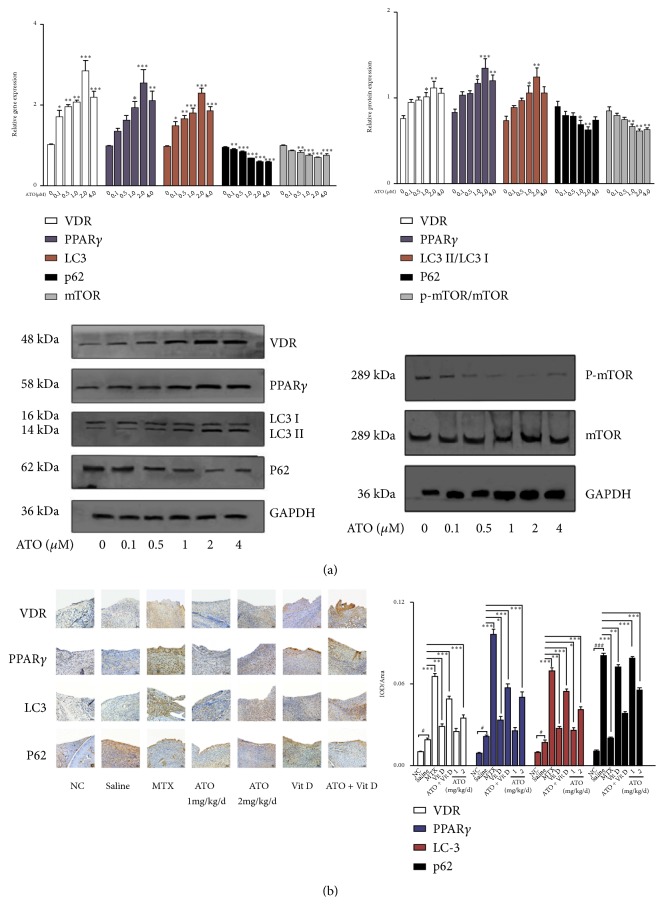
*Arsenic trioxide (ATO) and Vitamin D rescue the defective Vitamin D receptor (VDR)-PPAR-γ autophagy functional module in rheumatoid arthritis (RA)*. (a) Increased expression levels of PPAR-*γ*, VDR, and LC-3 and decreased expression levels of p62 after ATO administration in RA fibroblast-like synoviocytes (FLS) (*n*=3; *∗p*<0.05; *∗∗p*<0.01). Data are expressed as means ± standard error of the mean (SEM). (b) Increased average integrated optical density (IOD) values for VDR, PPAR-*γ*, LC-3, and p62 in synovial tissue in collagen-induced arthritis- (CIA-) saline mice (saline) compared with normal controls (NC). CIA mice under ATO and methotrexate (MTX) treatments showed additional increased average IOD values for VDR, PPAR-*γ*, and LC-3 but decreased p62 in synovial tissue compared with the saline group (*n*=6; *∗p*<0.05; *∗∗p*<0.01; *∗∗∗p*<0.001, experiment versus saline). Magnification = 20×. Bars = 50 *μ*m. Data are expressed as means ± SEM.

Regarding potential side effects and safety, we examined the influence of ATO and Vit D on the proliferation of RA FLS. Although the optical density value decreased, different concentrations of ATO and Vit D showed no significant influence on cell proliferation (Figure 1(a)). RA FLS were detected after treatment with different doses of ATO (0.1, 0.5, 1.0, 2.0, and 4.0 *μ*M). VDR, PPAR-*γ*, and LC-3 were significantly upregulated after ATO treatment, with the peak effect seen at 2 *μ*M; importantly, p62 was surprisingly significantly downregulated (Figure 2(a),* p*<0.05). Whether or not RA FLS exhibited defective autophagic flux was investigated by using VDR, PPAR-*γ*, LC-3, and p62 mRNA and protein in normal human (NH) FLS and RA FLS. The expression levels of VDR, PPAR-*γ*, and LC-3 were significantly upregulated in RA FLS compared with normal human FLS. Meanwhile, p62 mRNA and protein were also higher in RA FLS, which may indicate blockage of autophagy (Figure 1(b),* p *<0.05).

Afterwards, RA FLS were treated with ATO (2 *μ*M) after VDR knockdown. PPAR-*γ* and LC-3 were significantly downregulated but p62 was enhanced after silencing VDR, and ATO could reverse these effects (Figure 1(c),* p*<0.05). Immunohistochemistry was performed to validate the effect of ATO on the VDR-PPAR-*γ* autophagy functional module in CIA mice. Strikingly, treatment with ATO (2 mg/kg/d) or MTX (2 mg/kg/week) led to significant increases in the expression of VDR, PPAR-*γ*, and LC-3 and a decrease in p62 compared with CIA-saline mice (Figure 2(b),* p*<0.05). Thus, ATO alone may activate the VDR-PPAR-*γ* autophagy functional module both* in vitro* and* in vivo*.

### 3.2. Combined Effect of ATO and Vit D on the VDR-PPAR-*γ* Functional Module of Autophagy

**Figure 3:**
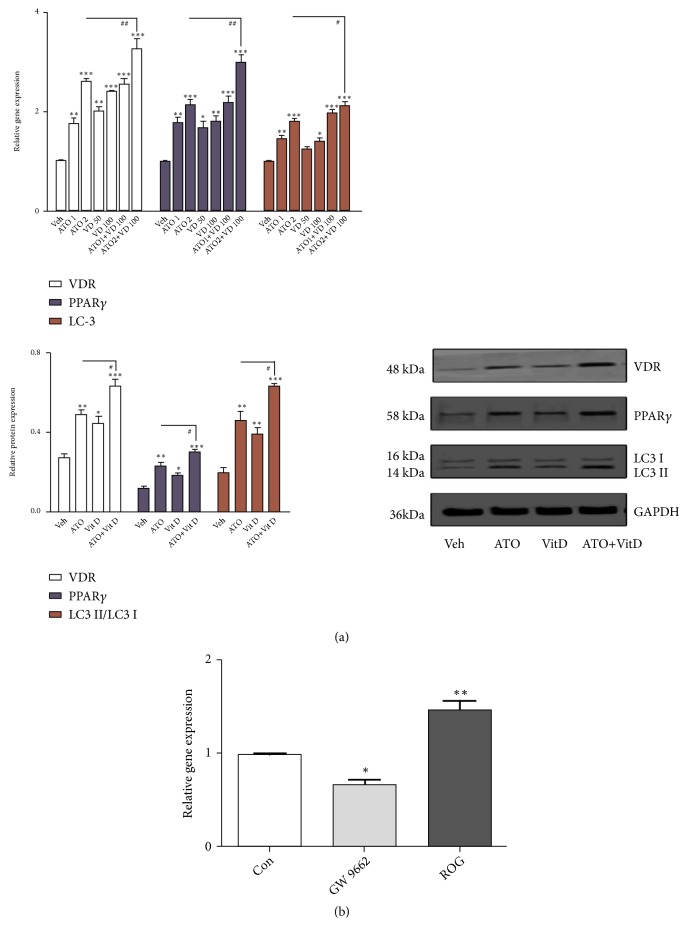
*Combined effect of arsenic trioxide (ATO) and Vitamin D (Vit D) on the Vitamin D receptor (VDR)-PPAR-γ autophagy functional module*. (a) Synergic upregulation of VDR, PPAR-*γ*, and LC-3 in rheumatoid arthritis (RA) fibroblast-like synoviocytes (FLS) after administration of ATO (2 *μ*M) and Vit D (50 and100 nM) alone or in combination compared with the vehicle (*n*=3; *∗p*<0.05; *∗∗p*<0.01; *∗∗∗p*<0.001). (b) Real-time PCR showed a significant decrease in the expression of VDR mRNA in RA FLS after treatment with GW9662, an antagonist of PPAR-*γ*. On the other hand, treatment with rosiglitazone (ROG) significantly upregulated VDR mRNA expression (*n*=3, *∗p*<0.05; *∗∗p*<0.01 versus RA FLS treated with the medium alone (control).

**Figure 4:**
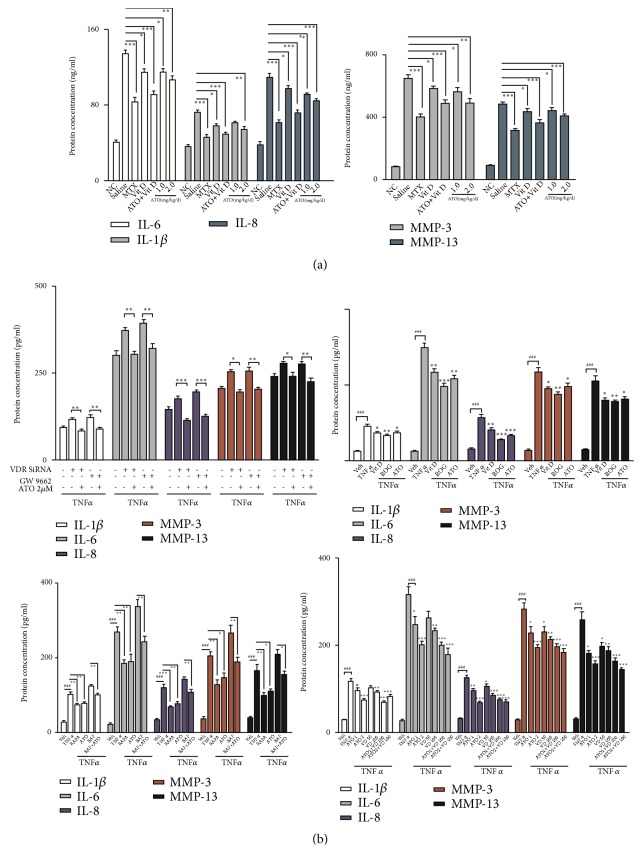
*Arsenic trioxide (ATO) suppresses the release of key inflammatory and catabolic cytokines in rheumatoid arthritis (RA)*. (a) Inflammation module representatives including interleukin-6 (IL-6), IL-1*β*, and IL-8 and catabolism module representatives including matrix metalloproteinase-3 (MMP-3) and MMP-13 decreased in mice serum after the administration of ATO compared with collagen-induced arthritis- (CIA-) saline, whereas they significantly increased in CIA-saline mice compared to normal controls (NC), as shown by enzyme-linked immunosorbent assays (ELISA) (*n*=6; *∗p*<0.05; *∗∗p*<0.01; *∗∗∗p*<0.001). (b) The administration of 2.0 *μ*M ATO significantly decreased the expression of inflammation module representatives and catabolism module representatives induced by tumor necrosis factor--*α* (TNF-*α*) (50 ng/mL) or by silencing the Vitamin D receptor (VDR) by small interfering RNA (siRNA) and inhibiting PPAR-*γ* with GW9662 (*n*=3, #*p*<0.05; ##*p*<0.01; ###*p*<0.001, vehicle versus TNF; *∗p* < 0.05; *∗∗p*<0.01; *∗∗∗p*<0.001, experiment versus TNF. Vehicle = RA fibroblast-like synoviocytes (FLS) with medium; TNF = RA FLS with TNF-*α* treatment alone). In addition, these cytokines were downregulated by rapamycin and upregulated by BafA1 but this was reversible by ATO (*n*=3, *∗∗p*< 0.05; *∗∗p*<0.01; *∗∗∗p*<0.001). ATO had synergistic effects with Vit D in downregulating the expression of IMRs and CMRs (*n*=3, #*p*<0.05; ##*p*<0.01; ###*p*<0.001, vehicle versus TNF; *∗p*<0.05; *∗∗p*<0.01; *∗∗∗p* <0.001, experiment versus TNF).

Both ATO and Vit D upregulated the expression of VDR, PPAR-*γ*, and LC-3* in vitro* (Figure 3(a),* p*<0.05), but ATO had a synergistic effect with Vit D on this upregulation in RA FLS (Figure 3(a),* p*<0.05); low doses of ATO (1 mg/kg/d) plus Vit D (400 ng/kg/d) achieved similar effects to ATO (2 mg/kg/d) (Figures 3(a) and 4(b)). Furthermore, ATO synergized with Vit D* in vivo* to upregulate the expression of VDR, PPAR-*γ*, and LC-3 significantly and downregulate p62 in the synovium of mice (Figure 2(b),* p*<0.05). Therefore, we consider that ATO may have a synergistic effect with Vit D on the VDR-PPAR-*γ* autophagy functional module* in vivo* and* in vitro*.

### 3.3. ATO Alleviates Symptoms and Joint Destruction in CIA Mice

**Figure 5:**
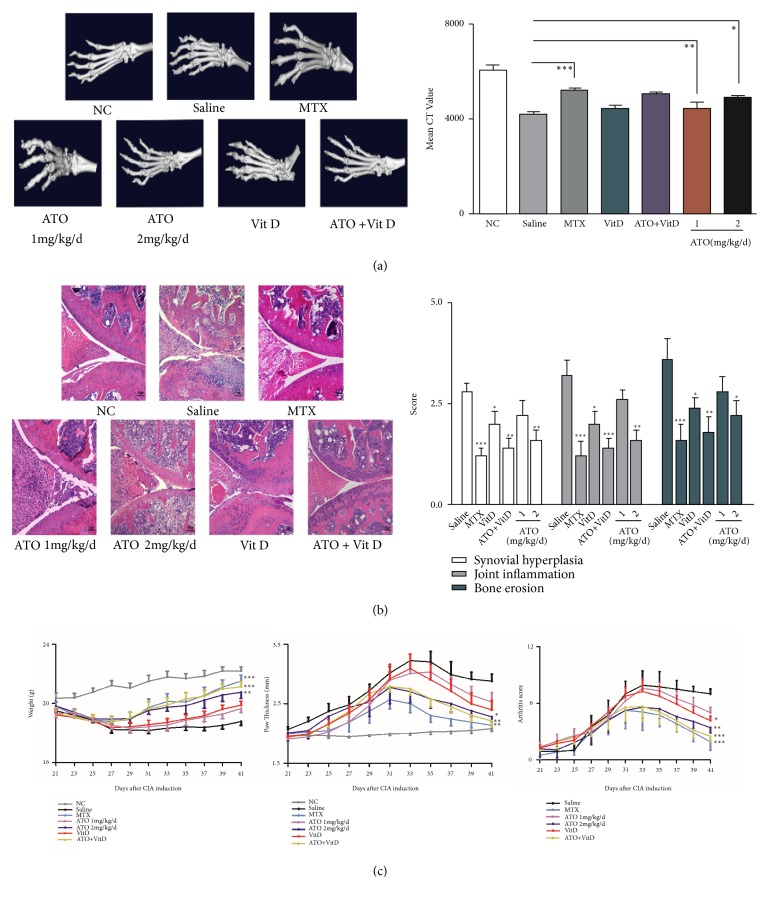
*Arsenic trioxide (ATO) alleviated the symptoms and joint destruction of CIA mice*. (a) ATO and Vitamin D (Vit D) alleviate joint destruction and significantly elevated mean CT values in collagen-induced arthritis (CIA) mice compared with normal control (NC) mice (*n*=6; *∗p*<0.05, *∗∗p*<0.01, *∗∗∗p*<0.001). Data are expressed as mean ± SEM. (b) The severity of arthritis in CIA mice was measured by H&E staining of knee joints. ATO treatment (2 mg/kg/d) and Vit D (400 ng/kg/d) significantly reduced the histological scores, including synovial hyperplasia, cartilage and bone erosion, and joint inflammation compared with NC (*n*=6; *∗p*<0.05, *∗∗p*<0.01, *∗∗∗p*<0.001). Magnification = 20×. Bars = 50 *μ*m. Data are expressed as mean ± SEM. (c) Collagen-induced arthritis- (CIA-) saline mice (saline) showed a significant decrease in weight and increased paw thickness and arthritis scores compared with the normal control (NC) group. Arsenic trioxide (ATO) at 2.0 mg/kg/day, Vitamin D (Vit D), and methotrexate (MTX) (n=6) significantly increased bodyweight and decreased paw thickness and arthritis scores (n=6, *∗*p<0.05, *∗∗*p<0.01, *∗∗∗*p<0.001, treatment versus saline). Data are expressed as means ± SEM.

We assessed the body weight, degree of paw swelling, and arthritis scores of the CIA and control mice. Both ATO (1 or 2 mg/kg/d) and Vit D (400 ng/kg/d) alone alleviated the symptoms of CIA mice (Figure 5(c),* p*<0.01). However, combining ATO and Vit D further reduced the arthritis score but this was not significantly different from ATO alone. The severity of arthritis in CIA mice was assessed by H&E staining of knee joint sections. ATO treatment at 2 mg/kg/d and Vit D (400 ng/kg/d) significantly reduced the histological scores including synovial hyperplasia, cartilage, and bone erosion and joint inflammatory (Figure 5(b),* n*=6,* p*<0.01). Additionally, ATO (2 mg/kg/d) significantly enhanced the mean CT values of hind paws compared with CIA-saline mice, as did MTX (2 mg/kg/w) (Figure 5(a);* n*=6,* p*<0.01).

### 3.4. ATO Inhibits the TNF-*α*-Induced Inflammation Module Representative and Catabolism Module Representative Release by Regulating the VDR-PPAR-*γ* Autophagy Functional Module

Interestingly, ATO (1 mg/kg/d) decreased MMP-13 expression, but key inflammation biomarkers such as IL-6, IL-1*β*, and IL-8 and catabolism factor module representatives including MMP-3 and MMP-13 were significantly suppressed by ATO (2 mg/kg/d) (Figure 4(a),* p*<0.01) but were significantly upregulated in CIA-saline mice compared with the normal control mice, as shown by ELISA (Figure 4(a),* p*<0.01). Vit D (400 ng/kg/d) alone significantly decreased these cytokines and may significantly enhance the effect of ATO when used in combination (Figure 4(a),* p*<0.05). Therefore, ATO and Vit D downregulated inflammation factors and catabolism factors in CIA mouse serum* in vivo*.

Furthermore, TNF-*α* treatment (50 ng/mL) significantly induced the inflammation factors and catabolism factors in RA FLS. Stimulation of the VDR-PPAR-*γ* functional module by its agonist Vit D and rosiglitazone (50 *μ*mol/L) decreased cytokine release, as did ATO with or without Vit D. In contrast, silencing VDR by siRNA and inhibiting PPAR-*γ* with GW9662 (10 *μ*mol/L) increased the secretion of inflammatory and catabolic factors (Figure 4(b),* p*<0.05); this was reversed by ATO. Furthermore, inflammation and catabolism factors were downregulated by stimulating autophagy with rapamycin (100 nmol/L). Consistently, the autophagy blocker BafA1 induced cytokine release but ATO reversed this effect (Figure 4(b),* p*<0.05). Therefore, ATO inhibited the release of TNF-*α*-induced inflammation factors and catabolism factors in RA FLS by regulating the VDR-PPAR-*γ* autophagy module.

## 4. Discussion

Autophagy is a highly conserved biological process in eukaryotic cells. Some autophagy-associated genes are closely related to RA [36].

Firstly, we explored the roles of VDR and PPAR-*γ* during autophagy in RA FLS. To the best of our knowledge, this is the first study reporting that the expressions of VDR, PPAR-*γ*, and LC-3 are significantly upregulated, and mTOR is significantly downregulated in RA FLS. Our results showed that p62 in RA FLS was significantly upregulated, indicating autophagic flux obstruction. Since impaired autophagic flux may be involved in a variety of diseases [37], we concluded that defective autophagic flux, rather than other factors causing functional loss of autophagy, leads to RA.

Indeed, PPAR-*γ* and VDR are interconnected and modulate the expression of genes in similar ways as a functional module [38, 39]. In order to further clarify the relationship between PPAR-*γ* and VDR in RA FLS, we applied rosiglitazone to excite PPAR-*γ* plaques in RA FLS and found that VDR expression was also elevated, suggesting that PPAR-*γ* may have a positive feedback effect on VDR.

Anti-TNF biological agents have been proven to be effective in the treatment of RA; however, around 30% of the patients respond poorly or not at all to the biologics. In addition, the treatment is limited by preexisting malignancy and inflammation and further impose an economic burden on patients because of the high costs. ATO has attracted global attention as one of several highly promising effective anticancer drugs. To our knowledge, this is the first report to demonstrate that ATO may rescue impaired autophagic flux beyond its role in activating the VDR-PPAR-*γ* autophagy functional module, consequently inhibiting the inflammatory response and joint destruction.

Previously, we and others reported that ATO may relieve the clinical symptoms of patients with leukemia and other rheumatic diseases [27, 40, 41]. Although we observed no significant effect of ATO on cell proliferation and no toxic effect on CIA mice, several adverse effects have been reported in patients such as liver injury and carcinogenesis [41], which limit the application of ATO in clinical settings. Solution could be sought regarding local drug delivery, drug combinations [42], or chemical modifications [43]. For the first time, we provided evidence that ATO showed a synergistic effect in combination with Vit D both* in vivo* and* in vitro*. Since Vit D is safe and easily available, the combination of the two drugs may reduce the dosage of ATO and mitigate its side effects.

## 5. Conclusion

In closing, VDR, PPAR-*γ*, LC-3, and p62 form a functional module, which is obviously upregulated in RA FLS and CIA mice synovial fibroblasts, but with impaired autophagic flux, as demonstrated by the upregulation of p62.

**Figure 6:**
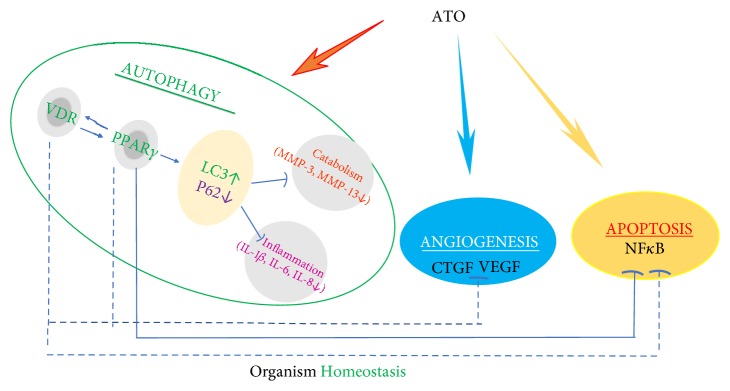
*Regulation effect of ATO on VDR-PPAR-γ functional module*. The Vitamin D receptor (VDR) and PPAR-*γ* interplay with each other and form a functional module. ATO rescued defective autophagy by regulating this functional module in synovial fibroblasts, thus inhibiting the expression of inflammatory and catabolic factors. Serving as a regulatory mechanism, autophagy has complex interrelationships with other physiological processes including angiogenesis and apoptosis, which can be regulated by ATO. The solid lines indicate our original data or results from our study that have been corroborated by other labs; dotted lines indicate that research is still ongoing.

Although the exact mechanisms are still poorly understood, ATO may enhance the VDR-PPAR-*γ* autophagy functional module and rescue impaired autophagic flux both* in vivo* and* in vitro* in RA and consequently inhibit the expression of inflammatory and catabolic factors that participate in joint destruction (Figure 6). Furthermore, we provided evidence that low-dose ATO showed a better effect in combination with Vit D in ameliorating RA symptoms, suggesting novel promising protocols for RA and cancer therapy.

## Figures and Tables

**Table 1:** Sequences of primers.

Target gene	Forward primer (5′–3′)	Reverse primer (5′–3′)
*GAPDH*	GCACCGTCAAGGCTGAGAAC	TGGTGAAGACGCCAGTGGA
*PPAR- γ*	TGGAATTAGATGACAGCGACTTGG	CTGGAGCAGCTTGGCAAACA
*VDR*	ACTCACCTCTGCCTCAATGTGAA	GATGAGGCAACAGCATTATCCAAG
*LC3*	CTTCTGAGCCAGCAGTAGGG	GGCAGAGTAGGTGGGTTGGT
*P62*	ACATAGCTTGCCTAATGGCTTTCAC	CCTGCCTGCTGACAACACCTA
*mTOR*	GGCCTGGATGGCAACTACAGA	TGACTGGCCAGCAGAGTAGGAA

## Data Availability

The data used to support the findings of this study are available from the corresponding author upon request.
